# Personality traits and motivations for mammography

**DOI:** 10.1192/j.eurpsy.2026.11548

**Published:** 2026-07-31

**Authors:** I. Chaari, A. Samet, W. Abid, Y. Fourti, F. Charfeddine, L. Aribi, N. Messedi, J. Aloulou

**Affiliations:** Psychiatric department “B”, Hedi Chaker University Hospital, Sfax, Tunisia

## Abstract

**Introduction:**

Mammography is a key tool for early breast cancer detection. While personality traits are known to affect health behaviors, their influence on women’s motivations for undergoing screening remains unclear.

**Objectives:**

To examine associations between personality traits and motivations for mammography.

**Methods:**

We conducted an online survey of the general female population in Tunisia via a sponsored Facebook campaign (December 2023–February 2024). Personality was assessed using the Big Five Inventory (BFI-44). Motivations were classified as preventive (routine screening) or non-preventive (fear, symptoms, family history).

**Results:**

A total of 278 women participated in the study, with a mean age of 46 years (SD 8.9, range 20–84). Most were married (84.9%), university educated (78.4%), and of middle socio-economic status (66.5%). Regarding the reasons for undergoing mammography, 62% did so for preventive purposes, whereas 38% cited anxiety or symptomatic concerns Preventively motivated women showed higher Conscientiousness scores (mean 3.9 vs 3.4, p = 0.04), while non-preventive women scored higher in Neuroticism (3.7 vs 3.1, p = 0.03). Logistic regression confirmed Conscientiousness as an independent predictor of preventive motivation (OR = 1.35, 95% CI 1.05–1.78, p = 0.02).

**Conclusions:**

In Tunisian women, conscientiousness supports preventive motivations, while Neuroticism drives anxiety-based motivations. Tailoring communication to personality traits may enhance participation in screening.

**Disclosure of Interest:**

None Declared

